# Impact of MyDiabetesPlan, a Web-Based Patient Decision Aid on Decisional Conflict, Diabetes Distress, Quality of Life, and Chronic Illness Care in Patients With Diabetes: Cluster Randomized Controlled Trial

**DOI:** 10.2196/16984

**Published:** 2020-09-30

**Authors:** Catherine Yu, Dorothy Choi, Brigida A Bruno, Kevin E Thorpe, Sharon E Straus, Paul Cantarutti, Karen Chu, Paul Frydrych, Amy Hoang-Kim, Noah Ivers, David Kaplan, Fok-Han Leung, John Maxted, Jeremy Rezmovitz, Joanna Sale, Sumeet Sodhi-Helou, Dawn Stacey, Deanna Telner

**Affiliations:** 1 St. Michael's Hospital (Unity Health Toronto) Toronto, ON Canada; 2 Department of Medicine Faculty of Medicine University of Toronto Toronto, ON Canada; 3 Dalla Lana School of Public Health University of Toronto Toronto, ON Canada; 4 Li Ka Shing Knowledge Institute St. Michael's Hospital (Unity Health Toronto) Toronto, ON Canada; 5 Department of Medicine University of Toronto Toronto, ON Canada; 6 Applied Health Research Centre Li Ka Shing Knowledge Institute St. Michael's Hospital (Unity Health Toronto) Toronto, ON Canada; 7 Southlake Regional Health Centre Newmarket, ON Canada; 8 Bridgepoint Active Healthcare (Sinai Health System) Toronto, ON Canada; 9 Mount Dennis Weston Health Centre Humber River Family Health Team Toronto, ON Canada; 10 Department of Family and Community Medicine, Women's College Hospital Toronto, ON Canada; 11 University of Toronto Toronto, ON Canada; 12 North York General Hospital Toronto, ON Canada; 13 Markham Stouffville Hospital Markham, ON Canada; 14 Sunnybrook Health Sciences Centre Toronto, ON Canada; 15 Musculoskeletal Health and Outcomes Research, Li Ka Shing Knowledge Institute, St. Michael's Hospital (Unity Health Toronto) Toronto, ON Canada; 16 Toronto Western Family Health Team Toronto General Hospital Research Institute, University Health Network Toronto, ON Canada; 17 School of Nursing, University of Ottawa Ottawa Hospital Research Institute Ottawa, ON Canada; 18 South East Toronto Family Health Team (Toronto East Health Network) Toronto, ON Canada

**Keywords:** shared decision making, goals of care, decision aid, diabetes mellitus, decisional conflict, quality of life, patient assessment of chronic illness care, diabetes distress, randomized clinical trials

## Abstract

**Background:**

Person-centered care is critical for delivering high-quality diabetes care. Shared decision making (SDM) is central to person-centered care, and in diabetes care, it can improve decision quality, patient knowledge, and patient risk perception. Delivery of person-centered care can be facilitated with the use of patient decision aids (PtDAs). We developed *MyDiabetesPlan*, an interactive SDM and goal-setting PtDA designed to help individualize care priorities and support an interprofessional approach to SDM.

**Objective:**

This study aims to assess the impact of *MyDiabetesPlan* on decisional conflict, diabetes distress, health-related quality of life, and patient assessment of chronic illness care at the individual patient level.

**Methods:**

A two-step, parallel, 10-site cluster randomized controlled trial (first step: provider-directed implementation only; second step: both provider- and patient-directed implementation 6 months later) was conducted. Participants were adults 18 years and older with diabetes and 2 other comorbidities at 10 family health teams (FHTs) in Southwestern Ontario. FHTs were randomly assigned to *MyDiabetesPlan* (n=5) or control (n=5) through a computer-generated algorithm. *MyDiabetesPlan* was integrated into intervention practices, and clinicians (first step) followed by patients (second step) were trained on its use. Control participants received static generic Diabetes Canada resources. Patients were not blinded. Participants completed validated questionnaires at baseline, 6 months, and 12 months. The primary outcome at the individual patient level was decisional conflict; secondary outcomes were diabetes distress, health-related quality of life, chronic illness care, and clinician intention to practice interprofessional SDM. Multilevel hierarchical regression models were used.

**Results:**

At the end of the study, the intervention group (5 clusters, n=111) had a modest reduction in total decisional conflicts compared with the control group (5 clusters, n=102; −3.5, 95% CI −7.4 to 0.42). Although there was no difference in diabetes distress or health-related quality of life, there was an increase in patient assessment of chronic illness care (0.7, 95% CI 0.4 to 1.0).

**Conclusions:**

Use of goal-setting decision aids modestly improved decision quality and chronic illness care but not quality of life. Our findings may be due to a gap between goal setting and attainment, suggesting a role for optimizing patient engagement and behavioral support. The next steps include clarifying the mechanisms by which decision aids impact outcomes and revising *MyDiabetesPlan* and its delivery.

**Trial Registration:**

ClinicalTrials.gov NCT02379078; https://clinicaltrials.gov/ct2/show/NCT02379078

## Introduction

Person-centered care, whereby health care providers are encouraged to partner with patients to co‐design and deliver personalized care [1], is critical to delivering high-quality diabetes care [2]. Shared decision making (SDM) is central to person-centered care [3] and can be facilitated with the use of patient decision aids (PtDAs), such as Diabetes Medication Choice [4] or patient activation programs [5]. A meta-analysis of 16 studies using SDM in diabetes care found an association with improved decision quality, patient knowledge, and patient risk perception [6]. Decisional conflict is a measure of decision quality and reflection of the individual’s uncertainty in choosing an option [7]. Decisions with low decisional conflict have been associated with less regret [8] and less emotional and psychological distress [9]. Thus, engagement of the person in the decision-making process to make a high-quality decision is an important step in delivering person-centered care.

Other markers of person-centered care in diabetes management include health-related quality of life [10], diabetes distress [11], and perception of chronic illness care [12]. Health-related quality of life reflects the combined impact of an individual’s physical, psychological, and social well-being on health-related quality of life, and in patients living with diabetes, lower quality of life is associated with poorer clinical outcomes such as glycemic control [13]. Decisional conflict is a reflection of an individual’s uncertainty in choosing an option, whereas diabetes distress refers to an individual’s emotional state as a result of the burden of self-care tasks related to diabetes self-management and is associated with reduced self-care, reduced quality of life, and poor glycemic control [11,14]. Similarly, an individual’s positive perception of chronic illness care is associated with improved self-management behaviors and glycemic control [12]. Thus, these outcomes reflect person-centered care.

### Decision Aids to Support Person-Centered Care

Delivery of person-centered care and optimization of these outcomes can be facilitated with the use of PtDAs. A systematic review of 105 studies found that PtDAs improved decision quality and process and reduced decisional conflict but had no impact on quality of life [15]. For trials evaluating PtDAs for diabetes decisions, patients were more likely to change their medication. With the evolution of technology, PtDAs have expanded from static pamphlets, booklets, or videos to include interactive video- and computer-based programs; the latter enable individualized content tailored to the patient’s characteristics and needs [16,17].

Given the complexity of diabetes care and multiple competing priorities, decision aids that support goal setting are particularly relevant. To date, one randomized controlled trial (RCT) of a goal-setting and SDM aid (which offered individually tailored risk information and treatment options for multiple risk factors to help patients prioritize between clinical issues) in 344 patients with uncomplicated type 2 diabetes found no difference in patient empowerment for setting and achieving goals [18]. However, this intervention neither addressed patient-important priorities and preferences specifically nor used a provider-specific point-of-care tool at the time of consultation.

### Objectives

To address this gap, we developed *MyDiabetesPlan* and a multicomponent PtDA toolkit, which includes patient-directed, provider-directed, and point-of-care tools. *MyDiabetesPlan* is an interactive SDM and goal-setting PtDA designed to help individualize care priorities and support an interprofessional approach to SDM, in the context of complex guideline recommendations for patients with type 1 or type 2 diabetes and other comorbidities. The overall aim of this study was to assess the impact of *MyDiabetesPlan* on decisional conflict, diabetes distress, health-related quality of life, and patient assessment of chronic illness care in individual patients in primary care practice groups randomized to *MyDiabetesPlan*.

## Methods

### Research Program Overview

We previously reported on how the development and refinement of *MyDiabetesPlan*, an interprofessional shared decision-making (IPSDM) toolkit, following the principles of user-centered design [19,20]. In this paper, we describe our assessment of the effectiveness of *MyDiabetesPlan* through a two-step cluster RCT followed by individual interviews. We used the Consolidated Standards of Reporting Trials (CONSORT) checklist (CONSORT-eHealth [21] and CONSORT extension for cluster trials [22]) to report this paper (Multimedia Appendix 1).

### Study Design

The study protocol and methods are described in previous studies [19,23]. We conducted a two-step, parallel, cluster RCT with a 1:1 allocation ratio. We selected a clustered design and randomized at the level of primary care practice groups to avoid contamination (eg, clinicians using an IPSDM approach with control patients). In brief, the first step was provider-directed (*MyDiabetesPlan* was delivered to physicians, nurses, dietitians*,* or pharmacists), whereas the second step (occurring 6 months later) was provider- and patient-directed (patients were asked to use *MyDiabetesPlan* by themselves before the appointment; this was then reviewed by the provider team). We chose a two-step approach because a prior feasibility study [20] identified that patients required clinician assistance for completing their initial *MyDiabetesPlan*. Outcome measures were administered at the first step (baseline), second step (6 months later), and follow-up (12 months later).

### Setting and Participants

All primary care practice groups in Southern Ontario that had interprofessional staff (eg, nurse, dietitian, or pharmacist) and electronic medical records (EMRs, to identify patients with diabetes) were invited to participate via email, telephone, and in-person or virtual presentation to the executive or medical director; groups without interprofessional staff or EMRs were excluded. All primary care physicians in these group practices were invited to participate. A research coordinator identified patients with diabetes (type 1 or type 2) and 2 other comorbidities (heart disease, stroke, hypertension, cancer, chronic lung disease, arthritis, inflammatory bowel disorders, and urinary incontinence) from each consenting physician’s practice using a combination of keywords, International Classification of Diseases and billing codes. Patients were excluded if they did not speak English, had documented cognitive deficits, were unable to provide informed consent, had limited life expectancy (<1 year), or were not available for follow-up. Potentially eligible patients were identified via EMR query, and eligibility was further confirmed by chart review; from this group, participants were randomly selected and invited to participate and provided consent by telephone.

### Intervention

*MyDiabetesPlan* was described previously [19]. It is a web-based PtDA in which patients populate their cardiometabolic and psychosocial profiles and general care priorities; *MyDiabetesPlan* then generates individualized diabetes-specific goals and strategies based on these inputs that the patients then select, resulting in an action plan. After randomization, at study onset, clinicians at intervention sites underwent a one-on-one 60-min tutorial in their clinic room by the research coordinator, with access to a one-page *how-to* guide and 2-min video. During subsequent clinical encounters, a member of the interprofessional team (nurse or dietitian) logged into *MyDiabetesPlan* and completed it with the patient; the physician subsequently reviewed the resultant action plan with the patient. At 6 months, patients at intervention sites were provided with a patient-directed *how-to* guide and video and directed to update *MyDiabetesPlan* according to their progress before the appointment. The research coordinator followed up with participants by email and telephone at study onset, followed by quarterly debriefing sessions, in both individual and group formats.

### Control

Clinicians in the control sites received paper copies of the executive summary of the Diabetes Canada clinical practice guidelines [24] and a postcard outlining web-based clinical information resources. After 6 months, patients in the control sites received a Diabetes Canada patient education pamphlet [25] regarding diabetes self-management and a postcard outlining web-based additional patient resources.

### Outcome Measures

The primary outcome was decisional conflict [26]; secondary outcomes were diabetes distress [27], health-related quality of life [28], chronic illness care [29], and intention to engage in IPSDM [30] (Table 1). Decisional conflict was measured using the Decisional Conflict Scale (DCS), which can predict individuals’ intentions and subsequent behavior. It has a test-retest coefficient of 0.81 and internal consistency coefficients ranging from 0.78 to 0.92 [7]. These outcomes were assessed at the individual participant level, at baseline, and at 6 months and 12 months (after an appointment) through a web-based survey or by mail.

**Table 1:** Outcome measures and validated scales.

Outcome		Scale	Description and psychometric properties
**Patient outcomes**			
	Decisional conflict	DCS^a^ (16-item, 5 subscales; O’Connor, 1995) [7]	This scale consists of 16 items with 5 response categories (0=strongly agree, 4=strongly disagree), where higher scores indicate greater decisional conflict. The scale includes subscales for uncertainty, informed, values clarity, support, and effective decision. Test-retest correlation and Cronbach alpha exceed .78. It correlated with related constructs of knowledge, regret, and discontinuance and had excellent predictive validity. A clinically significant effect size is 0.30 to 0.40; scores lower than 25 are associated with implementing decision; scores exceeding 37.5 are associated with decision delay or feeling unsure about implementation. The primary outcome decisional conflict has been demonstrated to be responsive to change over time and thus will yield meaningful results when measured at baseline and throughout the study intervention.
	Diabetes distress	DDS^b^ (Polonsky et al, 2005) [27]	The DDS is a 17-item instrument that assesses emotional distress and functioning specific to living with diabetes. Responses are scored on a 6-point Likert-type scale from 1=no problem to 6=serious problem. Scores can range from 17 to 102, with higher scores indicating poorer diabetes-related quality of life and lower scores indicating better diabetes-related quality of life. This instrument has been found to have high internal reliability with a Cronbach alpha of .93, good convergent validity with the CESD^c^ (*r*=0.56) and self-care behaviors including lower adherence to eating recommendations (*r*=0.30) and lower levels of physical activity (*r*=0.20).
	Health-related quality of life	SF^d^-12 (Ware, 1996) [31]	The SF-12 is a 12-item version of the SF-36. The SF-12 is a widely used and validated generic measure of health-related quality of life. It is a multidimensional measure of perceived health, assessing physical functioning, physical role, bodily pain, general health, vitality, social functioning, emotional role, and mental health. Scores ranges from 0 to 100, with higher scores reflecting better health. Its validity was demonstrated in studies of patients with various chronic conditions [32,33].
	Chronic illness care	PACIC^e^ Scale (Glasgow et al, 2005) [29]	The PACIC Scale assesses the degree to which care is congruent with the Chronic Care Model from the perspective of the patient. Specifically, it was designed to measure patient activation, goal setting, problem solving/contextual counseling, delivery system design/decision support, and follow-up/coordination. The PACIC Scale has been used to evaluate a variety of chronic health conditions, including type 2 diabetes [29,34,35]. It has moderate test-retest reliability (*r*=0.58 during the course of 3 months) and correlates moderately with measures of primary care and patient activation (*r*=0.32-0.60, median=0.50, *P*<.001).
**Clinician outcome**			
	Intention to engage in IPSDM^f^	CPD^g^ Reaction Questionnaire (Legare et al, 2014) [36]	This 11-item questionnaire is based on the Theory of Planned Behavior, encompassing instrumental attitude, affective attitude, subjective norm, and perceived behavioral control. It has a reliability that ranges from 0.67 to 0.93 [37]

^a^DCS: Decisional Conflict Scale.

^b^DDS: Diabetes Distress Scale.

^c^CESDS: Center for Epidemiological Studies Depression Scale.

^d^SF: Short Form.

^e^PACIC: Patient Assessment of Chronic Illness Care.

^f^IPSDM: interprofessional shared decision-making.

^g^CPD: Continuing Professional Development.

### Sample Size Calculation

With at least 40 patients per physician, 50% patient participation rate, and an anticipated patient attrition rate of 25% [38], we estimated that approximately 15 patients per practice would be able to participate. On the basis of a previous study using the DCS [7], we selected a standardized effect size of 0.4 with an SD of 0.6, α of .05, and β of .10. Previous data have shown that ρ (intraclass coefficient) for decisional conflict for patients living with diabetes clustered within primary care physicians is 0.013 [39]. Therefore, accounting for clustering, 56 patients per intervention/control group, or 4 sites per intervention/control group would be required.

### Randomization

Practices were simultaneously randomized and allocated by a biostatistician to either intervention or control using computer-generated randomization in a 1:1 ratio. Each practice was given a code, and the biostatistician was blinded to the allocation status. After assignment, investigators, research coordinators, and trial participants were no longer blinded to group allocation owing to the nature of the intervention. The list of all eligible patients from each cluster was randomly ordered; patients were recruited from this list until the target sample size was met.

### Data Collection

The practice and sociodemographic characteristics of clinicians and patients were obtained at baseline. Outcome data were collected using participant questionnaires [23] distributed through web or by mail according to patient preference for pragmatic reasons at baseline, 6 months, and 12 months.

### Analysis

A modified intent-to-treat analysis was conducted. For the primary and secondary outcomes, a linear mixed effect model was used to analyze the total score for each scale where site was the random effect with adjustment for baseline value. To account for missing data, we conducted a fully adjusted mixed effect model using repeated measurements [40-42]. The baseline variables we adjusted for were baseline DCS score, age, sex, ethnicity, educational attainment, employment, and living arrangements as well as a history of cancer and heart, musculoskeletal, respiratory, mental health, kidney, eye, and nerve disease.

The impact of sociodemographic variables on these outcomes (decisional conflict, diabetes distress, health-related quality of life, and chronic illness care) was assessed. Specifically, we fit a main effects model that adjusted for age, sex, ethnicity, education, employment status, and living arrangements as well as a fully adjusted model that included all interactions between treatment and the preceding variables. As these subgroup analyses were explorative, these were performed on the complete case data because the use of repeated measure data would necessitate the need for interactions with time as well. *P* values for the treatment effect in the baseline adjusted models used Satterthwaite approximation for the denominator degrees of freedom, whereas the tests of interactions (subgroup effects) used likelihood ratio tests from a full maximum likelihood estimation [43]. Irrespective of the test result on subgroups, the treatment effects were then shown by subgroup, estimated from the second model specified above, along with 95% CI and a *P* value that tested each interaction in this fully adjusted model. Descriptive statistics were used to describe *the intervention effect by MyDiabetesPlan* use. Analysis of variance was used to assess differences in the clinician’s intention to practice IPSDM scores between the intervention and control groups at baseline, 6 months, and 12 months. Analysis was performed in R version 3.5.2 [44], and the packages lme4 (version 1.1-21) and lmerTest (version 3.1-0) [43] were used to fit and report the mixed effect models.

### Research Ethics

The study was approved by the Research Ethics Boards of Bridgepoint Health (15-009-BP), Markham Stouffville Hospital (Canadian Institutes of Health Research [CIHR] protocol, v1, January 2013), Michael Garron Hospital (609-1410-Mis-245), North York General Hospital (13-0265), Southlake Regional Health Centre (0055-1314), St. Michael’s Hospital (13-014; includes Humber River Regional Hospital), Sunnybrook Health Sciences Health Centre (345-2013), University Health Network (16-6044), and Women’s College Hospital (2013-2058, 2014-0043-B).

### Funding

The study was funded by the CIHR Knowledge to Action Operating Grant (Funding reference number KAL 290086), which had no role in the design, collection, management, analysis or interpretation of data, or in the writing or publication of the manuscript. SS is supported by a Tier 1 Canada Research Chair. NI was supported by New Investigator Awards from CIHR and from the Department of Family and Community Medicine, University of Toronto.

## Results

### Setting and Participants

**Figure 1:**
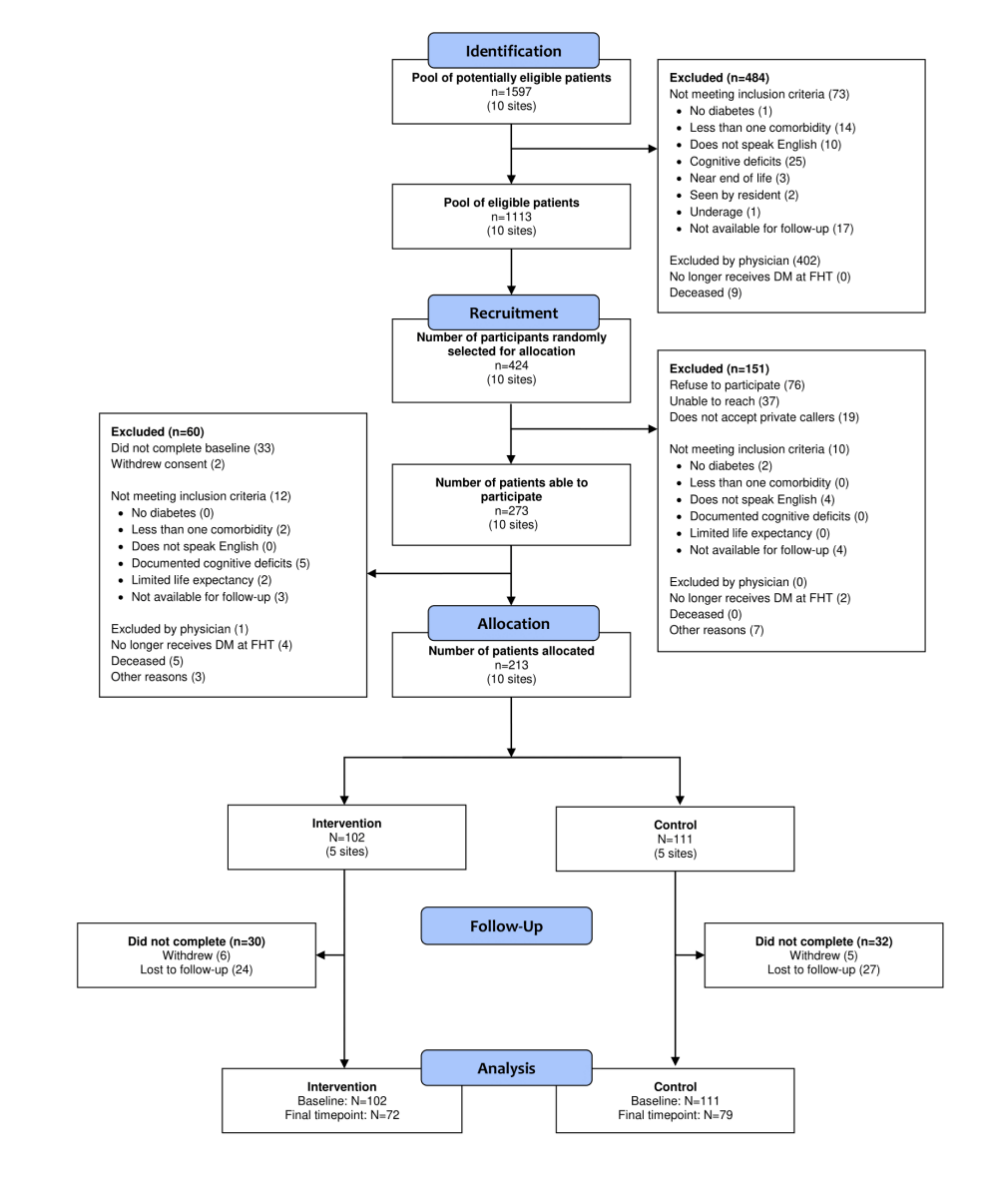
CONSORT (Consolidated Standards of Reporting Trials) flow diagram. DM: diabetes mellitus; FHT: family health team.

A total of 10 primary care practice groups were recruited from December 2014 to November 2015; patients were recruited from December 2015 to September 2016, followed by October 2016 to September 2017. Recruitment metrics and the CONSORT flow diagram are shown in Figure 1. The practice and sociodemographic characteristics of clinicians and patients are shown in Table 2. In the intervention group, 50.0% (51/102) and 46% (33/72) of participants completed the questionnaire via web-based platform for time points 1 and 3, respectively. In the control group, 47.7% (53/111) and 51% (40/79) of participants completed the questionnaire via web-based platform for time points 1 and 3, respectively (Multimedia Appendix 1).

**Table 2:** Clinician and patient characteristics.

Characteristics				Control, n (%)		Intervention, n (%)
**Clinician**				24 (100)		29 (100)
	**Sex at birth**					
		Female	11 (46)		21 (72)	
		Male	10 (42)		7 (25)	
		Prefer not to answer	3 (12)		1 (3)	
	**Duration in practice (years)**					
		2-5	7 (29)		5 (17)	
		6-10	5 (21)		8 (28)	
		≥11	12 (50)		16 (55)	
	**Number of patients with diabetes seen per week**					
		<10	12 (50)		17 (59)	
		≥10	12 (50)		10 (34)	
		Unsure	0 (0)		2 (7)	
	**Type of health team**					
		Community	20 (83)		5 (17)	
		Academic	4 (17)		24 (83)	
**Patients**				111 (100)		102 (100)
	**Age (years)**					
		18-44	7 (6.3)		2 (2.0)	
		45-54	9 (8.1)		11 (11.0)	
		55-64	28 (25.2)		20 (20.0)	
		65-74	38 (34.2)		47 (47.0)	
		75-84	24 (21.6)		16 (16.0)	
		≥85	5 (4.5)		4 (4.0)	
	**Sex at birth**					
		Female	46 (41.4)		56 (54.9)	
		Male	65 (58.6)		46 (45.1)	
	**Language**					
		English	103 (92.8)		81 (81.0)	
		Other	8 (7.2)		19 (19.0)	
	**Ethnicity**					
		White	75 (67.6)		62 (63.3)	
		Black	8 (7.2)		5 (5.1)	
		Asian	8 (7.2)		19 (18.6)	
		Indigenous	3 (2.7)		4 (4.1)	
		Latin American	2 (1.8)		1 (1.0)	
		Other	15 (13.5)		7 (7.1)	
	**Education**					
		Bachelor’s	17 (16.0)		23 (23.2)	
		Below bachelor	5 (4.7)		3 (3.0)	
		College	26 (24.5)		30 (30.3)	
		High school	31 (29.2)		19 (19.2)	
		Postgraduation	12 (11.3)		13 (13.1)	
		Below high school	15 (1.2)		11 (11.1)	
	**Employment**					
		Retired	63 (58.3)		54 (55.1)	
		Full time with employee health benefits	15 (13.9)		22 (22.4)	
		Full time/part time without employee health benefits	8 (7.4)		8 (8.2)	
		Government assistance/disability	6 (5.6)		3 (3.1)	
		Unemployed	5 (4.6)		2 (2.0)	
		Stay-at-home parent, student, volunteer	5 (4.6)		2 (2.0)	
		Other	4 (3.7)		7 (7.1)	
		Prefer not to answer	2 (1.9)		0 (0.0)	
	**Income, Can $ (US $)**					
		<10,000 (7603)	9 (8.7)		6 (7.6)	
		10,000-19,000 (7603-14,446)	18 (17.5)		6 (7.6)	
		20,000-29,000 (15,206-22,048)	8 (7.8)		5 (6.3)	
		30,000-39,000 (22,809-29,651)	13 (12.6)		7 (8.9)	
		40,000-49,000 (22,543-37,254)	10 (9.7)		7 (8.9)	
		50,000-59,000 (38,015-44,857)	8 (7.8)		5 (6.3)	
		60,000-69,000 (45,617-52,460)	3 (2.9)		6 (7.6)	
		70,000-79,000 (53,220-60,063)	6 (5.8)		6 (7.6)	
		80,000-89,000 (60,823-67,666)	2 (1.9)		6 (7.6)	
		90,000-99,000 (68,426-75,269)	8 (7.8)		6 (7.6)	
		100,000-149,000 (76,029-113,283)	7 (6.8)		8 (10.1)	
		≥150,000 (114,044)	11 (10.7)		11 (13.9)	
	**Living arrangements**					
		Alone	30 (27.3)		26 (25.7)	
		With family members	24 (21.8)		28 (27.7)	
		With partner/spouse	46 (41.8)		38 (37.6)	
		With roommates	2 (1.8)		3 (3.0)	
		Other	8 (7.3)		6 (5.9)	

### Attrition Analysis

A total of 62 patients withdrew or were lost to follow-up. Of these patients, we had demographic data on 34 patients (control, n=10; intervention, n=24; Multimedia Appendix 1). There were proportionately more non–English-speaking patients (9/34, 26%) with high school education (11/34, 32%) who withdrew, compared with those who remained in the study.

### Primary Outcome: Decisional Conflict

Total decisional conflict was modestly reduced in the intervention group at 12 months compared with the control group (−3.5 of a total score of 100, 95% CI −7.4 to 0.4, *P*=.08; Table 3). At 12 months, the *Uninformed* subscale was reduced in the intervention group (−3.9, 95% CI −8.8 to −1.02, *P*=.11). Similarly, at 12 months, the *Unclear Values* subscale was reduced in the intervention group (−3.6, 95% CI −9.6 to 2.28, *P*=.21).

### Secondary Outcomes

#### Patient Chronic Care, Diabetes Distress, and Quality of Life

Patient assessment of chronic illness care increased in the intervention group compared with the control group (0.7 of a total score of 5, 95% CI 0.4 to 1.0, *P*<.001). There was a small difference in diabetes distress (−0.2, 95% CI −0.4 to 0.05, *P*=.12) and quality of life (1.2, 95% CI −3.2 to 5.5, *P*=.57; Table 3).

**Table 3:** Scores at baseline, 6 and 12 months, and treatment effect at 6 and 12 months for decisional conflict, patient assessment of chronic illness care, diabetes distress, and quality of life.

Outcome measures	Score, mean (SD)							Treatment effect				
	Control			Intervention				6 months			12 months	
	Baseline	6 months	12 months		Baseline	6 months	12 months		Mean, 95% CI	*P* value	Mean, 95% CI	*P* value
DCS^a^ (out of 100; higher score represents more decisional conflict)	23.56 (15.00)	21.10 (12.79)	19.58 (9.11)		25.53 (14.73)	21.97 (14.87)	17.35 (11.21)		−1.82, −6.02 to 2.38	.38	−3.49, −7.4 to −0.42	.08
PACIC^b^ (out of 5)	3.16 (0.95)	3.41 (1.05)	3.22 (1.08)		2.82 (1.10)	3.16 (1.10)	3.68 (0.99)		0.15, −0.19 to 0.50	.35	0.71, 0.38 to 1.04	<.001
DDS^c^ (out of 6)	1.93 (0.83)	1.88 (0.78)	1.90 (0.75)		2.08 (1.02)	1.92 (1.09)	1.86 (0.87)		−0.08, −0.34 to 0.18	.53	−0.18, −0.42 to 0.05	.12
Quality of Life (SF^d^-12; out of 100)	89.69 (12.48)	87.77 (12.87)	86.99 (10.69)		87.35 (14.25)	88.88 (13.56)	87.94 (12.87)		3.47, −1.05 to 7.98	.12	1.18, −3.18 to 5.54	.57

^a^DCS: Decisional Conflict Scale.

^b^PACIC: Patient Assessment of Chronic Illness Care.

^c^DDS: Diabetes Distress Scale.

^d^SF: Short Form.

#### Intervention Effect of MyDiabetesPlan

A total of 52 patients completed (eg, *generated a complete plan) MyDiabetesPlan* two or more times during the study period. The greatest reduction in decisional conflict occurred in patients who completed *MyDiabetesPlan* more than 2 times, whereas the smallest reduction in decisional conflict occurred in patients who completed it 2 times (Table 4).

**Table 4:** Change in decisional conflict over 12 months in the intervention group using MyDiabetesPlan by number of completed plans.

Completed plans, n	Participants, n (N=68)	Decisional conflict score, mean (SD)		
		0 months	12 months	Change in score
<1	6	23.4 (22.7)	14.9 (11.8)	−8.5 (22.4)
1	20	23.2 (12.9)	13.9 (11.6)	−9.3 (10.6)
2	29	26.9 (10.9)	21.2 (11.5)	−5.7 (12.5)
>2	13	32.1 (22.5)	19.8 (14.6)	−12.3 (20.9)

#### Subgroup Analyses

For decisional conflict, we found weak evidence for any interaction (*P*=.07) that appeared to be driven by age>65 years (*P*=.01; Multimedia Appendix 1). We found stronger evidence when we examined the “uninformed” subscale (*P*=.03), driven by age>65 years (*P*=.003), and income>Can $50,000 (US $379,000; *P*=.04). Similarly, for diabetes distress, there was weak evidence for interaction (*P*=.14) driven by age>65 years (*P*=.01) and unemployment status (*P*=.03). There was weak evidence for interactions for PACIC (*P*=.01; lives alone: *P*=.11). There was little evidence of discernible interactions for the Short Form-12.

#### Intention to Engage in IPSDM

There was little evidence of differences between the 2 groups of clinicians in intention to practice SDM (Multimedia Appendix 1).

#### Harms

There were no harms associated with participation in this study.

## Discussion

### Principal Findings

We found that *MyDiabetesPlan*, an interprofessional goal-setting decision aid for people living with diabetes, resulted in a modest reduction in decisional conflict (specifically the *uninformed* subscale) and increased patient assessment of chronic illness care but had no impact on diabetes distress or health-related quality of life. *MyDiabetesPlan* reduced decisional conflict and diabetes distress most prominently in participants older than 65 years. There was no impact of *MyDiabetesPlan* use on clinicians’ intention to practice SDM.

Our finding regarding the impact of *MyDiabetesPlan* on decisional conflict is generally consistent with the literature, although our results did not meet statistical significance. A 2017 systematic review found that decision aids reduced decisional conflict related to uncertainty caused by unclear values (−8.81/100; 95% CI −11.99 to −5.63; 23 studies; n=5068; high-quality evidence) and feeling uninformed (−9.28/100; 95% CI −12.20 to −6.36; 27 studies; n=5707; high-quality evidence) [45]. In our study, we found smaller reductions: a 3.5-point reduction in the total scale, a 3.9-point reduction in the *uninformed* subscale, and a 3.7-point reduction in the *unclear values* subscale. This discrepancy may be due to the goal-setting nature of *MyDiabetesPlan*: rather than offering risks and benefits of a single discrete decision, it offered risks and benefits of multiple potential strategies. Thus, answering a question in this context, such as “I am clear about which benefits matter most to me” may be more challenging.

### Comparison With Previous Work

Consistent with a previous review [45], we also found no impact of *MyDiabetesPlan* on general or condition-specific health-related quality of life such as diabetes distress (though there was a signal for an effect in participants older than 65 years). Authors have postulated that this may be owing to the fact that decision aids are often used in situations where the options have no clear health outcome advantage [45]. A subanalysis of this review identified 11 studies involving 2684 patients that examined the impact of PtDA on health-related quality of life [46]. Of the 11 trials, 6 trials neither reported difference in health-related quality of life between PtDA and control nor over time. This confirmed the lack of impact, suggesting that health-related quality of life may be an uninformative end point unless a specific hypothesis for its impact can be made [46]. Another potential reason for this is that the outcome is too distant from the act of using *MyDiabetesPlan*: patients may select a goal (such as increasing physical activity) but may not enact the goal (ie, *actually increasing their physical activity*), and *thus may not experience any change in health-related quality of life.* Although *MyDiabetesPlan* may have reduced decisional conflict and increased patient activation and thus increased goal setting, there may be a gap between goal setting and goal attainment; bridging this gap with additional behavioral supports to optimize goal attainment may then result in improvements in health-related quality of life.

Our finding that *MyDiabetesPlan* reduced decisional conflict and diabetes distress, particularly in participants older than 65 years, is consistent with the literature [47]. This systematic review of 22 studies examining the impact of PtDa in adults aged 65 years and older found that people exposed to a decision aid had greater knowledge (5 studies; mean difference 6.5, 95% CI 0.76 to 12.25) and reduced decisional conflict (11 studies; mean difference −3.17 out of 100, 95% CI −4.44 to 1.90). These findings are reassuring, particularly in light of concerns of social inequities and the digital health divide [48], and provide support that a digital health innovation such as *MyDiabetesPlan* is appropriate for a complex older population with multiple comorbidities.

The strengths of this study include rigorous adherence to RCT methodology (including central randomization, similar intervention and control groups at baseline, appropriate length of follow-up, use of validated scales, intention-to-treat analysis), its finding of reducing decisional conflict and improving chronic care delivery, especially in those older than 65 years, and its generalizability. We designed this study to assess *the feasibility of implementing MyDiabetesPlan* in interprofessional primary care settings; as such, it is primarily a pragmatic trial along the explanatory-pragmatic continuum [49]. Thus, our study results mainly reflect what would happen if *MyDiabetesPlan* would be implemented in the usual clinical practice of interprofessional primary care.

### Limitations

Study limitations include the lack of blinding of participants (patients and clinicians) owing to the nature of the intervention, use of both paper- and web-based data collection methods, attrition rate of 29%, and less-than-anticipated *MyDiabetesPlan* use, resulting in potential bias, and its lack of clinical outcome measures. Although different data collection methods may introduce respondent bias, recent literature has shown that response rates do not differ between paper- and web-based respondents [50] and that there was no difference in socioeconomic characteristics between paper- and web-based respondents [51]. Our attrition rate and reduced *MyDiabetesPlan* use were amplified by our complex study population, many of whom either withdrew owing to competing health concerns or were lost to follow-up, presumably for similar reasons. Challenges to conducting research in this population are well documented, and solutions for future studies include providing transportation compensation, conducting home visits, and encouraging greater engagement of family members [52]. Although we did not assess clinical outcome measures, we assessed proximal patient-reported outcomes appropriate for a decision-aid intervention, outcomes that have typically been underused in the literature [53], and outcomes that have been associated with clinical outcomes such as A_1c_ [12-14]. Assessment of researcher-selected clinical outcomes such as A_1c_ in a trial where patients are encouraged to select their own personalized goals is incongruent with the principles of person-centered outcomes research [54]. Future studies may consider the use of goal attainment scaling (which has some demonstrated validity evidence in the geriatric care setting) [55] or composite clinical outcomes, though this is not without its limitations [56].

### Conclusions

*MyDiabetesPlan* modestly reduced decisional conflict and increased patient assessment of chronic illness care but had no impact on diabetes distress or health-related quality of life. The next steps in this research program are to engage with knowledge users (patients and their caregivers, clinicians, managers, and policy makers) to discuss the implications of these findings, modify *MyDiabetesPlan* and its mode of delivery, and consideration of clinical, organizational, and health care system contexts to plan scale-up to an implementation trial.

## Appendix

Multimedia Appendix 1 Supplemental file including CONSORT (Consolidated Standards of Reporting Trials) checklists (cluster, eHealth) and supplemental Tables 1-4.

## Abbreviations

CIHR: Canadian Institutes of Health Research
CONSORT: Consolidated Standards of Reporting Trials
DCS: Decisional Conflict Scale
EMR: electronic medical record
FHT: Family Health Team
IPSDM: interprofessional shared decision-making
PtDA: patient decision aid
RCT: randomized controlled trial
SDM: shared decision making
